# The receptor for Granulocyte-colony stimulating factor (G-CSF) is expressed in radial glia during development of the nervous system

**DOI:** 10.1186/1471-213X-8-32

**Published:** 2008-03-27

**Authors:** Friederike Kirsch, Carola Krüger, Armin Schneider

**Affiliations:** 1 SYGNIS Bioscience, Im Neuenheimer Feld 515, 69120 Heidelberg, Germany

## Abstract

**Background:**

Granulocyte colony-stimulating (G-CSF) factor is a well-known hematopoietic growth factor stimulating the proliferation and differentiation of myeloid progenitors. Recently, we uncovered that G-CSF acts also as a neuronal growth factor in the brain, which promotes adult neural precursor differentiation and enhances regeneration of the brain after insults. In adults, the receptor for G-CSF is predominantly expressed in neurons in many brain areas. We also described expression in neurogenic regions of the adult brain, such as the subventricular zone and the subgranular layer of the dentate gyrus. In addition, we found close co-localization of the G-CSF receptor and its ligand G-CSF. Here we have conducted a systematic expression analysis of G-CSF receptor and its ligand in the developing embryo.

**Results:**

Outside the central nervous system (CNS) we found G-CSF receptor expression in blood vessels, muscles and their respective precursors and neurons. The expression of the G-CSF receptor in the developing CNS was most prominent in radial glia cells.

**Conclusion:**

Our data imply that in addition to the function of G-CSF and its receptor in adult neurogenesis, this system also has a role in embryonic neurogenesis and nervous system development.

## Background

Granulocyte colony-stimulating (G-CSF) factor is a secreted glycoprotein of 20 kDa traditionally known as a hematopoietic growth factor stimulating the proliferation and differentiation of myeloid progenitors [1,2]. It is clinically used for the treatment of chemotherapy-associated neutropenia and for the mobilization of stem cells for bone-marrow transplantations. In addition to this hematopoietic function, we recently described important functions in the central nervous system (CNS), including anti-apoptotic properties on mature neurons, as well as a neurogenic function in adult neural stem cells [3]. G-CSF and its receptor are widely expressed in the adult CNS and induced upon cerebral ischemia. Besides reducing infarct volumes in stroke, G-CSF enhances long-term recovery after insults to the brain, which is linked to an increase in neurogenesis [3-5]. Taken together, these data underline the clinical relevance of G-CSF as a potential new drug for stroke and other neurodegenerative disorders (for review see [6]).

G-CSF and its receptor show a broad, mainly neuronal, colocalized expression throughout the murine brain [3]. Beside the expression in pyramidal neurons in the cortex (mainly layers II/III and V), the Purkinje cell layer of the cerebellum, the hippocampus (hilus and CA3 field), the entorhinal cortex and the olfactory bulb, G-CSF is also expressed in neurogenic regions in the adult brain: in the subgranular zone of the dentate gyrus and the subventricular zone. To see whether the expression in the nervous system of the adult organism had any correlation to embryonic expression of the receptor, we performed an expression pattern study of the G-CSF receptor during CNS development of the mouse embryo.

## Results

### G-CSF receptor expression outside the CNS throughout development of the rat embryo

**Figure 1:**
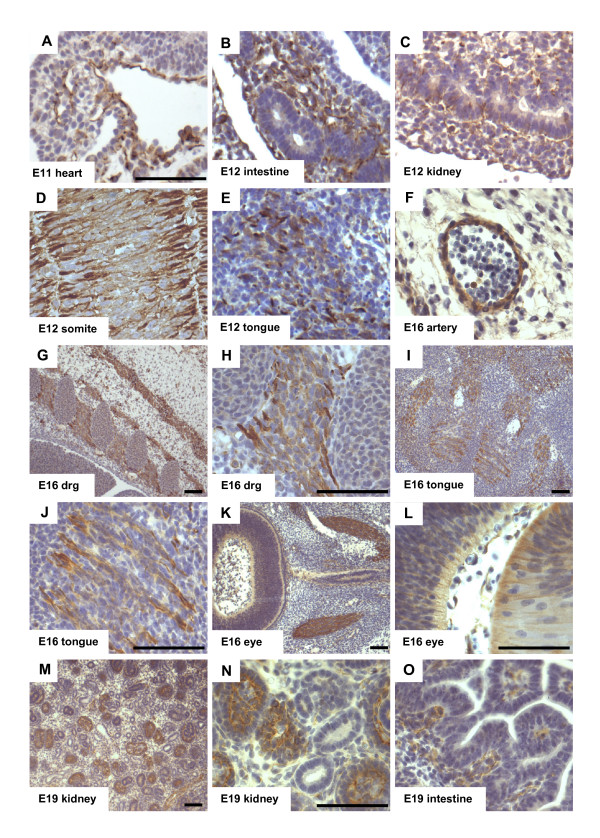
**The G-CSF receptor is expressed in structures outside the brain throughout development of the rat embryo**. The figure gives an overview of the G-CSF receptor expression pattern in the rat embryo. **A **E11 heart, **B **E12 intestine, **C **E12 kidney, **D **E12 somite, **E **E12 tongue, **F **E16 cerebral artery, **G, H **E16 cervical dorsal root ganglia, **I, J **E16 tongue, **K, L **E16 eye, **M, N **E19 kidney, **O **E19 intestine, (Immunohistochemical staining of 10 μm paraffin sections, scale bar 50 μm, drg: dorsal root ganglia, E: embryonic day)

In order to analyze the G-CSF receptor expression in the murine embryonic development, we performed immunohistochemical stainings. Figure 1 gives examples of G-CSF receptor expression outside the developing CNS from E11–E19. G-CSF receptor expression can be found in vessels of the cardiac ventricle (Figure 1A; E11), of the intestine (Figure 1B, O; E12 and E19), of glomeruli in the kidney (Figure 1C, M, N; E12 and E19) and in the wall of blood vessels (Figure 1F; E16). Moreover, the G-CSF receptor is expressed in neurons of the upper cervical dorsal root ganglia (Figure 1G, H; E16), in nerve fibers and muscles of the tongue (Figure 1E, I, J; E12 and E16) and in the developing retina (Figure 1L; E16). Receptor expression was also detected in muscle precursors (somite, Figure 1D; E12) and muscles (e.g. the external ocular muscles in the eye (Figure 1K; E16). In addition, its expression can be observed in the lens fibers of the eye (Figure 1L; E16).

### Identification of mRNA expression of the G-CSF receptor and G-CSF in the developing CNS

**Figure 2:**
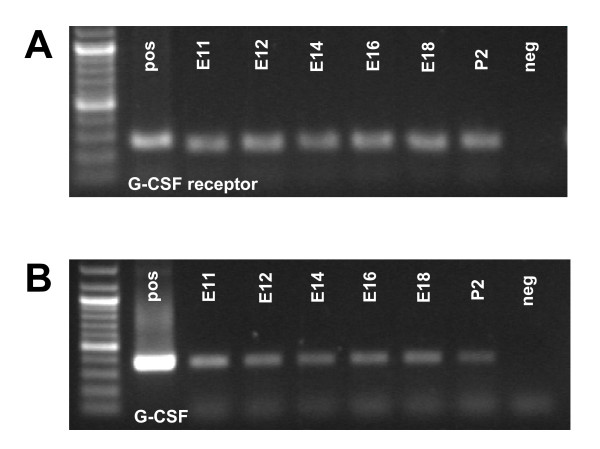
**Expression of G-CSF and its receptor on the RNA level**. G-CSF receptor (A) and its ligand G-CSF (B) are present throughout murine embryonic development, which is demonstrated by RT-PCR. RT-PCR products of the following stages were loaded on the gels: E11, E12, E14, E16, E18 and P2. As positive controls for G-CSF and G-CSF receptor sequence-verified cDNA clones were used. The negative control consisted of water. RNA was prepared out of E11 and E12 whole embryonic mouse heads, for the other stages mouse brain was taken. Primers are located in the 3' part of the open reading frames with product lengths of 235 bp for G-CSF receptor and 360 bp for G-CSF. (Pos., positive PCR control (G-CSF or G-CSF receptor, respectively; neg., negative control)

Next we analyzed the G-CSF receptor expression in the developing CNS. We detected expression of the G-CSF receptor and G-CSF during murine embryonic CNS development by reverse transcriptase PCR (RT-PCR). In heads or brains of embryo development stages E11 – E18 and postnatal day 2 (P2, Figure 2) PCR yielded the expected product sizes (235 bp for G-CSF receptor and 360 bp for G-CSF). G-CSF and its receptor are detected as early as E11, and are expressed continuously throughout P2.

### Immunohistochemical localization of the G-CSF receptor in the developing CNS

**Figure 3:**
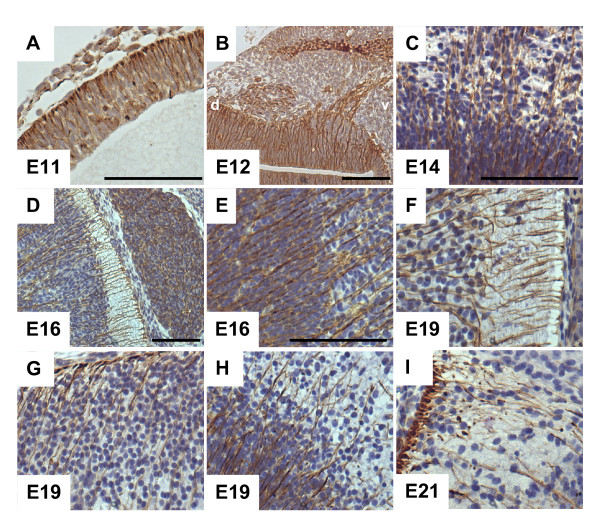
**G-CSF receptor is expressed in the embryonic nervous system**. The expression shows characteristics of radial glia cells in terms of long processes and termination in end-feet. **A **E11 forebrain, **B **E12 spinal cord with dorsal root ganglion, axon root and muscle, **C **E14 hindbrain, **D **E16 spinal cord with dorsal root ganglion, **E **E19 spinal cord, **F **E19 spinal cord, **G **E19 hindbrain, **H **E21 olfactory bulb, **I **E21 diencephalon, (Immunohistochemical staining of 10 μm paraffin sections, scale bar = 50 μm, d: dorsal, E: embryonic day, v: ventral).

By immunohistochemistry, the RNA signal of the G-CSF receptor at E11 corresponds to protein expression in the telencephalic vesicle of the E11 embryo (Figure 3A). Later in development we found G-CSF receptor expression in other CNS regions, for example in the developing spinal cord (Figure 3B, D, E; E12 and E16), in the hindbrain (Figure 3C, G; E16, E19), in the olfactory bulb (Figure 3H; E19), in the diencephalon (Figure 3I; E21) and in the retina (Figure 1L, E16). Cells with G-CSF receptor expression commonly displayed long and fine processes, which pass through the neuroepithelial layer and terminate in small end feets at the pial surface (e.g in Figure 3A). This morphology matches exactly that described for radial glia cells by Ramón y Cajal [7]. During postnatal development the radial glia scaffold decreases and the G-CSF receptor is expressed in developing neurons (Additional file 1).

In addition, we found G-CSF receptor expression in structures of the peripheral nervous system such as the dorsal root ganglion and axon roots originating from the ventral spinal cord (Figure 3B; E12).

### G-CSF receptor and Nestin co-localize in the E16 rat embryo

**Figure 4:**
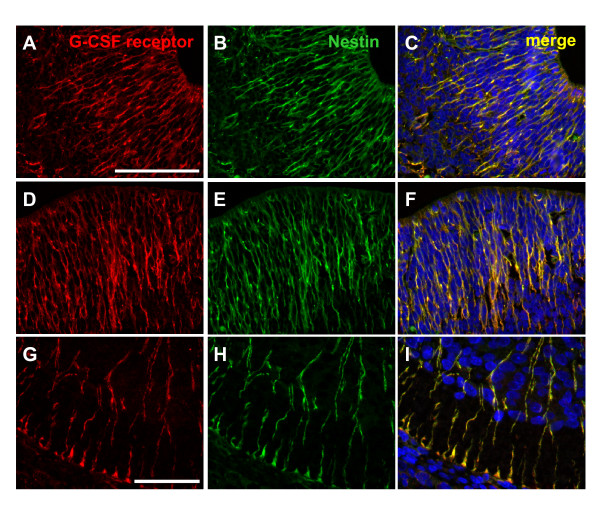
**G-CSF receptor and Nestin co-localize in the nervous system of the E16 rat embryo**. The G-CSF receptor staining is shown in red, the signal for Nestin green. On images **A-C **the staining was performed on cortex tissue, **D-F **diencephalon and **G-I **spinal cord tissue. **C, F **and **I **are merged images.(Immunofluorescent staining on 10 μm paraffin sections, scale bar 50 μm, E: embryonic day).

To further prove that the structures which express the G-CSF receptor in the developing nervous system are radial glia cells, we performed co-immunofluorescence labeling with the stem cell marker Nestin, which labels radial glia cells at these embryonic stages (e.g. [8]). Figure 4 shows a complete overlap of G-CSF receptor and Nestin signals in cortex (Figure 4A–C), diencephalon (Figure 4D–F) and spinal cord (Figure 4G–I) tissue.

## Discussion

The embryonic expression pattern of the G-CSF receptor can be divided into three main categories. The first category with prominent G-CSF receptor expression consists of blood vessels (e.g. the cardiac ventricles, in the intestine, in the glomerular system of the kidney and in blood vessels in the CNS (Figure 1A–C, 1F, 1M–O). Expression in endothelial cells in the embryo is expected as the G-CSF receptor is expressed in adult blood vessels, and G-CSF has considerable effects on endothelial cell functions [9-11]. Expression in blood vessels in the brain likely forms the basis for blood-brain barrier penetration of G-CSF [3,12], and for effects on G-CSF-induced vascular remodeling after stroke [13,14] or myocardial infarction [15].

The second category with G-CSF receptor expression is formed by muscles and their progenitors (e.g. somites and ocular muscle, Figure 1D, K).

Neurons represent the third category, where high abundance of G-CSF receptor expression was observed in the rat embryo (e. g. in the tongue, the upper cervical dorsal root ganglia, Figure 1E, 1G–J). Broad neuronal expression of G-CSF receptor and its ligand have been detected in the adult CNS [3,16]. Using RT-PCR, we detected embryonic expression of G-CSF receptor and G-CSF from E11 up to postnatal day 2 in the brain, respectively head of mice, consistent with the immunohistochemical detection. The embryonic expression pattern of the G-CSF receptor in the CNS is characterized by staining of elongated radial processes crossing through the developing neuroepithelium (Figure 3) and terminating in conical endfeet at the the pial surface (Figure 3A, G, I). We identified these cells as radial glia because of their unique morphology and co-expression of the intermediate filament protein Nestin (initially described as monoclonal antibody Rat-401) [17,18]. Because of their long processes, radial glia cells were originally interpreted as a transient scaffold upon which nascent neurons should migrate to their definitive positions [19,20]. In recent years, the view of radial glia cells as pure supportive cells has changed dramatically. They are now regarded as multifunctional cells involved in many aspects of brain and spinal cord development. One important feature of radial glia might be the involvement in CNS regionalization. In hindbrain development for example, it has been shown that rhombomere boundaries are formed by radial glia cells in order to limit neuronal migration [21]. Most intriguing however, is the recent perception that radial glia cells are the neuronal progenitors that generate neurons in the cerebral cortex [22,23]. The remaining adult neural stem cells found for example in the subventricular zone of the lateral ventricle in the rodent forebrain are derived from the embryonic radial glial population [24-26]. This strong relationship between radial glia and adult stem cells is further highlighted by the fact that adult NSCs can redifferentiate to radial glia [27]. The finding that the G-CSF receptor is found on radial glial cells is therefore fully concordant with our data on the later expression of the receptor on adult neural stem cells [3,28].

## Conclusion

Implications from our finding are threefold: First, it adds a further dimension to the presence of the GCSF system in the nervous system (i.e. embryonic expression and a likely significance early in development of the nervous system). Second, it underlines the link between radial glial cells and adult neural stem cells where G-CSF has functional relevance. Third, our data provide a new and highly specific marker (G-CSFR) for radial glial cells throughout cortical neurogenesis.

## Methods

### Immunohistochemistry

Wistar rat embryos were fixed in 4% paraformaldehyde and embedded in paraffin. 10 μm paraffin sections were deparaffinized and microwaved in citrate buffer. For G-CSF receptor staining sections were blocked in 1% H_2_O_2 _in TBS for 30 minutes at room temperature. After washing, incubation with the G-CSF receptor antibody (Santa Cruz Biotechnology, Inc., sc-9173, 1:100) followed overnight at 4°C in a humid chamber. Staining was visualized after washing and secondary antibody incubation (anti-rabbit biotin, Dianova) using the avidin-biotin complex (ABC) technique with DAB as chromogen (DakoCytomation). In order to visualize the nuclei, sections were treated with haemalaun. The specifity of the G-CSF receptor staining is demonstrated by omission of the primary antibody (Additional file 2).

For double immunofluorescence with Nestin, the sections were blocked in 0,2% BSA/TBS followed by antibody incubation at 4°C overnight (anti-mouse nestin, Chemicon International, MAB353, 1:100) and G-CSF receptor antibody. Subsequent washing, sections were incubated for detection with the appropriate fluorescent dye-conjugated secondary antibodies (anti-mouse FITC and anti-rabbit Cy5, Dianova, 1:200). Nuclear staining was performed using Hoechst33342.

### RT-PCR

RNA of mouse brains (E14, 16, 18, P2) and mouse heads (E11, 12) respectively was isolated using phenol-chloroform extraction [29] followed by QIAGEN RNeasy Mini Kit purification according to the manufacturer's recommendations. cDNA was synthesized from 5 μg total RNA using oligo-dT primers and Superscript III Reverse Transcriptase (Invitrogen Corp.). The following primers were used for RT-PCR: mouse G-CSF-790s, GGA GCT CTA AGC TTC TAG ATC; mouse G-CSF-1154as, TAG GGA CTT CGT TCC TGT GAG; mouse G-CSF receptor-2582s, TGT GCC CCA ACC TCC AAA CCA; mouse G-CSF receptor-2817as, GCT AGG GGC CAG AGA CAG AGA CAC. Cycling conditions were as follows for G-CSFR: 5 minutes at 95°C, 30 seconds at 95°C, 30 seconds at 60°C, 30 seconds at 72°C for 34 cycles. The final extension time was 7 minutes at 72°C. For G-CSF, the same program was used after 2 cycles of a touchdown program from 64°C–62°C.

## Authors' contributions

AS conceived of the study, FK and CK conducted experiments and analyzed data, AS and FK wrote the manuscript. All authors read and approved the final manuscript.

## Supplementary Material

Additional file 1 **During postnatal development the radial glia scaffold decreases and the G-CSF receptor is expressed in emerging neurons**. The figure shows a G-CSF receptor immunostaining at postnatal day 7. The decreasing radial glia scaffold is marked by arrows. (10 μm paraffin sections, scale bar 50 μm) Click here for file

Additional file 2 **Control for G-CSF receptor immunostaining specificity**. The figure demonstrates a control for staining specificity of the G-CSF receptor by omission of the primary antibody. **A**, E17, spinal cord, G-CSF receptor immunostaining, **B**, control with omission of the primary antibody. (Immunofluorescent staining on 10 μm paraffin sections, scale bar 50 μm, E: embryonic day). Click here for file
