# Supplementary figures and images for: The receptor for Granulocyte-colony stimulating factor (G-CSF) is expressed in radial glia during development of the nervous system
Source: BMC Dev Biol. 2008 Mar 27;8:32. doi: 10.1186/1471-213X-8-32 (PMC2329616; doi:10.1186/1471-213X-8-32)

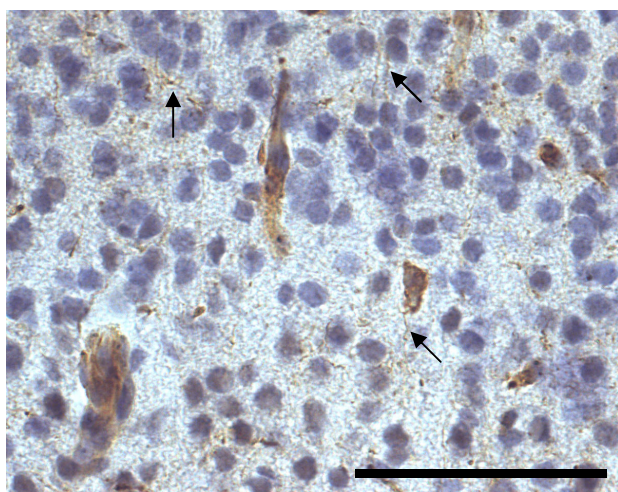

**Additional file 1**

Supplement: Additional file 1 — During postnatal development the radial glia scaffold decreases and the G-CSF receptor is expressed in emerging neurons. The figure shows a G-CSF receptor immunostaining at postnatal day 7. The decreasing radial glia scaffold is marked by arrows. (10 μm paraffin sections, scale bar 50 μm) [file 1471-213X-8-32-S1.pdf]

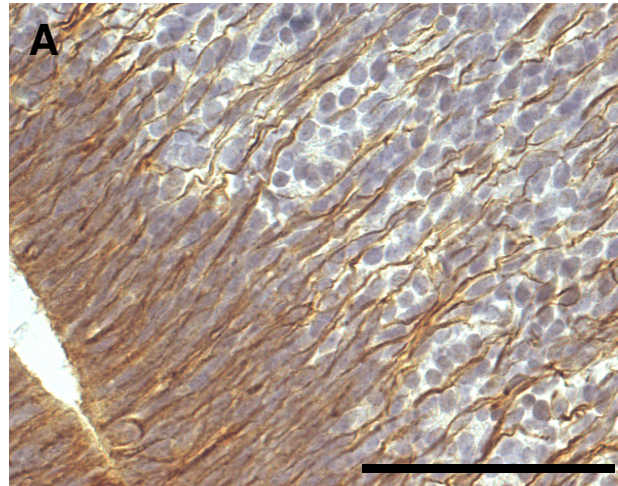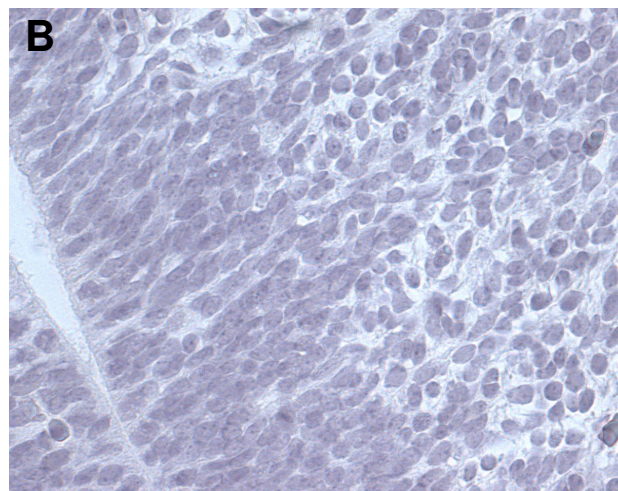

**Additional file 2**

Supplement: Additional file 2 — Control for G-CSF receptor immunostaining specificity. The figure demonstrates a control for staining specificity of the G-CSF receptor by omission of the primary antibody. A, E17, spinal cord, G-CSF receptor immunostaining, B, control with omission of the primary antibody. (Immunofluorescent staining on 10 μm paraffin sections, scale bar 50 μm, E: embryonic day). [file 1471-213X-8-32-S2.pdf]
